# Draft Genome Sequence of Fusobacterium vincentii CNGBCC1850030, Isolated from Healthy Human Feces

**DOI:** 10.1128/mra.00543-22

**Published:** 2023-03-23

**Authors:** Xiaohuan Jing, Chuanfa Liu, Yuman Ye, Jingchun Xu, Hongmei Huang, Bo Wang, Jinpu Wei, Jiao Zhao

**Affiliations:** a China National GeneBank, BGI Shenzhen, Shenzhen, China; b BGI Shenzhen, Shenzhen, China; c College of Wildlife Resources, Northeast Forestry University, Xiangfang District, Harbin, China; Wellesley College

## Abstract

Fusobacterium vincentii usually inhabits the oral cavity and plays an important role in periodontal diseases. Here, we report the draft genome sequence of *F. vincentii* strain CNGBCC1850030, isolated from healthy human feces.

## ANNOUNCEMENT

Fusobacterium vincentii is a Gram-negative, obligately anaerobic fusiform bacterium (1–3). Fusobacterium nucleatum subsp. *vincentii* was first detected by Vincent in 1896 in lesion tissue samples taken from patients with acute necrotizing ulcerative gingivitis and Vincent’s angina (4). Fusobacterium nucleatum subsp. *vincentii* was reclassified as Fusobacterium vincentii in 2021 (1). *F. vincentii* may play an important role in the initiation and progression of periodontal disease (5, 6). Here, we describe the genome sequence of Fusobacterium vincentii CNGBCC1850030, which was isolated from healthy human feces (from a 30-year-old man, without any clearly diagnosed chronic or malignant disease) collected in Shenzhen (Guangdong Province, China). The sample collection was approved by the Bioethics Committee of CNGB (CNGB-IRB), and the approval number is CNGB_irb1705.

To isolate single microbial strains, 1.0 g of the sample was suspended in 10.0 mL of stroke physiological saline solution (0.9%). Serial dilutions of up to 10^−6^ were prepared, and 100-μL aliquots were plated on brain heart infusion agar and incubated in an anaerobic glove box (90% N_2_, 5% CO_2_, and 5% H_2_) at 37°C for 48 h. The isolated single colony was identified as the species Fusobacterium vincentii by 16S rRNA gene sequencing. The pure colony was cultured and stored at −80°C in 20% glycerol (vol/vol) containing 0.1% cysteine. For genome sequencing, CNGBCC1850030 was grown overnight in 50 mL brain heart infusion broth. The cell suspension was then centrifuged at 12,000 rpm/min, and the pellet was collected and used for DNA extraction with a DNA extraction kit (Magen; catalog number MD5115-02B; MagPure stool DNA KF kit B).

The genomic DNA was fragmented to 300 bp to 500 bp using a sonicator (Covaris, USA). Then, a 100-bp paired-end (PE100) DNA library was prepared using the easy universal DNA library prep set (MGI, China). Sequencing was performed on the BGISEQ-500RS platform with a BGISEQ-500RS high-throughput sequencing set (PE100) (MGI), and 10,488,936 read pairs were generated. The reads were filtered to remove adapter sequences and low-quality sequences using bbduk.sh with default settings using the BBTools version 38.95 suite (https://sourceforge.net/projects/bbmap/); sequencing errors were corrected using Lighter version 1.1.2 (7). The quality of the reads was checked using FastQC (8). The filtered reads were assembled using assembly-scan version 0.4.1 (https://github.com/rpetit3/assembly-scan) and Shovill version 1.1.0 (https://github.com/tseemann/shovill) with default settings. The final assembly contained 2,078,835 bp in 32 contigs with a G+C content of 26.97%. The maximum contig size was 425,627 bp, and the *N*_50_ value was 107,676 bp. To assess the completeness of the genome assembly, lineage-specific marker gene sets were analyzed using CheckM version 1.1.3 (9). This analysis showed 100.00% completeness, 0.00% contamination, and 0.00% heterogeneity. The assembled genome contained 1,907 genes (coding DNA sequences [CDSs]), 3 rRNAs, 46 tRNAs, and 3 noncoding RNAs (ncRNAs), as annotated using the Prokaryotic Genome Annotation Pipeline (PGAP) version 5.1 of the National Center for Biotechnology Information (10, 11). The average nucleotide identity with the closest strain, Fusobacterium nucleatum subsp. *vincentii* ATCC 49256 (GenBank assembly accession number GCA_000182945.1) was 97.62% using dRep version 3.0.0 (with -p 20 -pa 0.97 -sa 0.99).

### Data availability.

This genome project was deposited at NCBI. The NCBI BioProject accession number is PRJNA847373, and the BioSample accession number is SAMN28932599. The raw data files can be obtained from the NCBI SRA under accession number SRR19592273. The genome sequence can be found at GenBank under accession number JAMXTT000000000.
